# Atmospheric pathway of marine heatwaves over the Northwestern Pacific

**DOI:** 10.1038/s41598-023-49833-4

**Published:** 2023-12-20

**Authors:** El Noh, Joowan Kim, Sang-Yoon Jun, Gyundo Pak, Joo-Hong Kim, Hyeong-Gyu Kim

**Affiliations:** 1 Department of Atmospheric Science, Kongju National University, Gongju, South Korea; 2 Earth Environment Research Center, Kongju National University, Gongju, South Korea; 3 Korea Polar Research Institute, Inchon, South Korea; 4 Korea Institute of Ocean Science and Technology, Pusan, South Korea

**Keywords:** Atmospheric science, Ocean sciences

## Abstract

This study analyzes the influence of the Pacific–Japan (PJ) atmospheric teleconnection pattern and its interaction with oceanic processes on sea surface warming over the Northwestern Pacific. The PJ pattern is a thermally driven Rossby wave that originates over the tropical western Pacific through deep convection and propagates toward high latitudes. It plays a significant role in sea surface warming by inducing anticyclonic circulation and the corresponding northwestward extension of the subtropical high over the Northwestern Pacific. This study revealed that the key processes responsible for sea surface warming were an increase in insolation and a decrease in the ocean-to-atmosphere latent heat flux under the anticyclonic conditions driven by the PJ. This finding provides valuable insights into the role of atmospheric processes, we refer to it as the “*atmospheric pathway*”, in the development of East Asian marine heatwaves (MHWs). A detailed understanding of this process will contribute to the prediction and mitigation of MHWs in East Asian countries.

## Introduction

Under the global warming over land and ocean (IPCC AR6^1^), the frequency and intensity of extreme heat events have been increasing in major cities (heatwave^2–4^) and coastal regions (marine heatwave, MHW^5–7^) with projections of growing likelihood^8,9^. Heatwaves have severe impacts on human health and society, often posing the risk of fatality^10^, and MHWs have detrimental effects on marine environments^11,12^. In particular, MHWs have devastating effects on coastal and ocean ecosystems^13^ as well as the fishery industry^7,14^. The need to predict MHWs to mitigate and minimize their adverse impacts on ocean ecosystems and the fishery industry is increasing^5,15^. However, the accurate prediction of these phenomena remains challenging because of their irregular behavior and lack of understanding^16^.

The East Asian coast is vulnerable to strong MHWs owing to the warm ocean conditions created by the Kuroshio Current^16,17^. Moreover, a large amount of heat exchange between the ocean and atmosphere occurs in this region^18^; thus, the East Asian coast and Northwestern Pacific are subject to potential sea surface warming by air-sea interactions^19,20^. For instance, the record-breaking MHW event that occurred over the East Asian coast in 2016 was closely related to abnormally high air-sea heat fluxes into the ocean^21,22^. Recently, Lee et al.^14^ proposed two major modes of East Asian MHWs, based on contrasting sea surface temperature patterns. One of the MHW modes corresponds with the large-scale atmospheric patterns that cause East Asian atmospheric heatwaves^23–26^. In particular, the atmospheric teleconnection pattern that propagates meridionally along the East Asian coast, known as the Pacific–Japan (PJ) pattern, shows significant coherence with atmospheric heatwaves and MHWs over Korea and Japan^26–28^.

The PJ pattern is caused by atmospheric Rossby waves originating from tropical convection over the western Pacific^29,30^ and plays a significant role in weather and climate variability in East Asia during boreal summer^31,32^. Recently, many studies have focused on the relationship between PJ patterns and near-surface warming over East Asia, which often develops into heatwaves^23,26,27,33,34^. Shimpo et al.^33^ showed that a major factor in the 2018 heatwave was the continued expansion of the surface Northwestern Pacific subtropical high (NPSH) and upper tropospheric Tibetan high toward East Asia with the assistance of the PJ pattern. Using quantitative energy budget analysis, Noh et al.^26^ revealed that horizontal heat and moisture advection associated with the PJ pattern is the dominant process for near-surface warming in East Asia. Overall, the PJ pattern and associated quasi-stationary Rossby waves along the East Asian coast significantly contribute to the onset of heatwaves over East Asia^35^ and are recognized as key predictors for subseasonal forecasting over East Asia, particularly for heatwaves occurring in boreal summer^34^.

Here, we report that the atmospheric PJ pattern could significantly increase the likelihood of MHWs over the Northwestern Pacific, which experiences strong heating events^22,36,37^ and consequential ecological impacts^17^ (e.g., 2021 MHW case). This study aimed to elucidate the physical processes driving MHWs. We first examined sea surface warming along the East Asian coast during negative PJ events and performed a quantitative analysis using the mixed layer heat budget to identify the causes of the warming. The overall analyses are based on composites of the 39 negative PJ events that are detected for 43 years (1979–2021) and provide robust results. The results discover that the sea surface warming is caused by shortwave and latent heat flux changes owing to the atmospheric circulation associated with the PJ pattern. These results suggest that atmospheric teleconnection patterns could serve as important processes driving MHWs over the East Asian coast on intraseasonal timescales.

## Result

**Figure 1:**
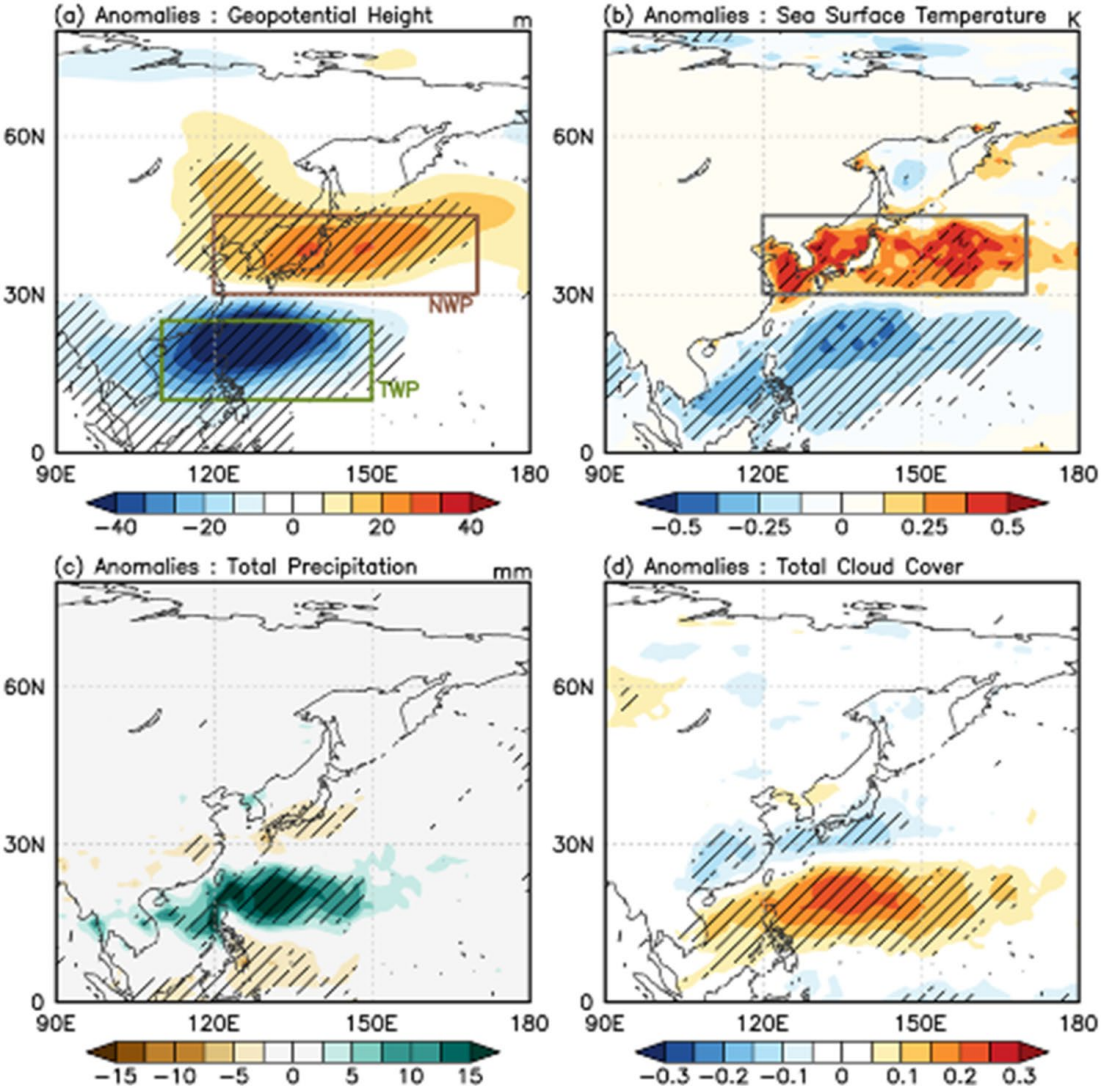
Composite maps of (**a**) 850-hPa geopotential height (m), (**b**) sea surface temperature (K), (**c**) total precipitation (mm), and (**d**) total cloud cover anomalies for the negative Pacific–Japan patterns. The dashed areas denote statistically significant anomalies at 95% confidence level based on a two-tailed Student’s *t*-test. The map information used in this study was obtained from the data provided by the NCAR Command Language (NCL).

Overall features of the atmospheric response during negative PJ events are summarized in composite maps along with the sea surface temperature anomalies (Fig. 1). The spatial pattern of 850-hPa geopotential height anomalies shows a clear meridional dipole structure (Fig. 1a). This structure can be explained by atmospheric Rossby waves that originate from the tropical western Pacific (TWP; green box in Fig. 1a) and propagate poleward^29,30^. The cyclonic anomalies over the TWP coincide with enhanced deep convection caused by moisture convergence from the Indian Ocean and tropical Pacific during the negative PJ event^38^ (refer to Supplementary Fig. S4). This enhanced deep convection was well recognized with increased total precipitation and total cloud cover over the tropical western Pacific region (Fig. 1c,d). The organized convection act as source of the Rossby waves^29^, and southwesterly winds of the NPSH provide a wave guide for the propagation^32,39^.

The meridionally propagating Rossby waves affect weather conditions in mid-latitude. The most prominent feature is the high-geopotential (high-pressure) anomaly over the Northwestern Pacific (NWP; brown box in Fig. 1a). The high-pressure anomaly coheres well with anticyclonic circulation anomalies in this region and typically supports the northwestward expansion of the NPSH^27,40^. This high-pressure system also reduces the cloud cover (Fig. 1d) and supports near-surface warming by enhancing insolation. Several studies have investigated the high-pressure related atmospheric warming over East Asia^26,27,35^, and recent study revealed that horizontal temperature advection and increased insolation associated with the high-pressure system are the key drivers of the near-surface atmospheric warming^26^.

The intriguing, but still unknown feature is the sea surface temperature (SST) response during PJ events. The SST anomalies during the negative PJ (Fig. 1b) demonstrates a clear dipole pattern, particularly with a warm anomaly over the NWP. The mid-latitude warming pattern extends widely from the Yellow Sea to the region of the Kuroshio Extension, and this signal is robustly found also in buoy-based in-situ SST observations (Supplementary Fig. S1a). Several studies have reported the relationship between PJ and sea surface warming^14,37,41–43^. For example, the large-scale atmospheric pattern observed in the 2021 record-breaking MHW demonstrates an anomalous meridional dipole over the East Asia like Fig. 1a^37,43,44^. However, key processes driving the sea surface warming during negative PJ remain incompletely understood.

**Figure 2:**
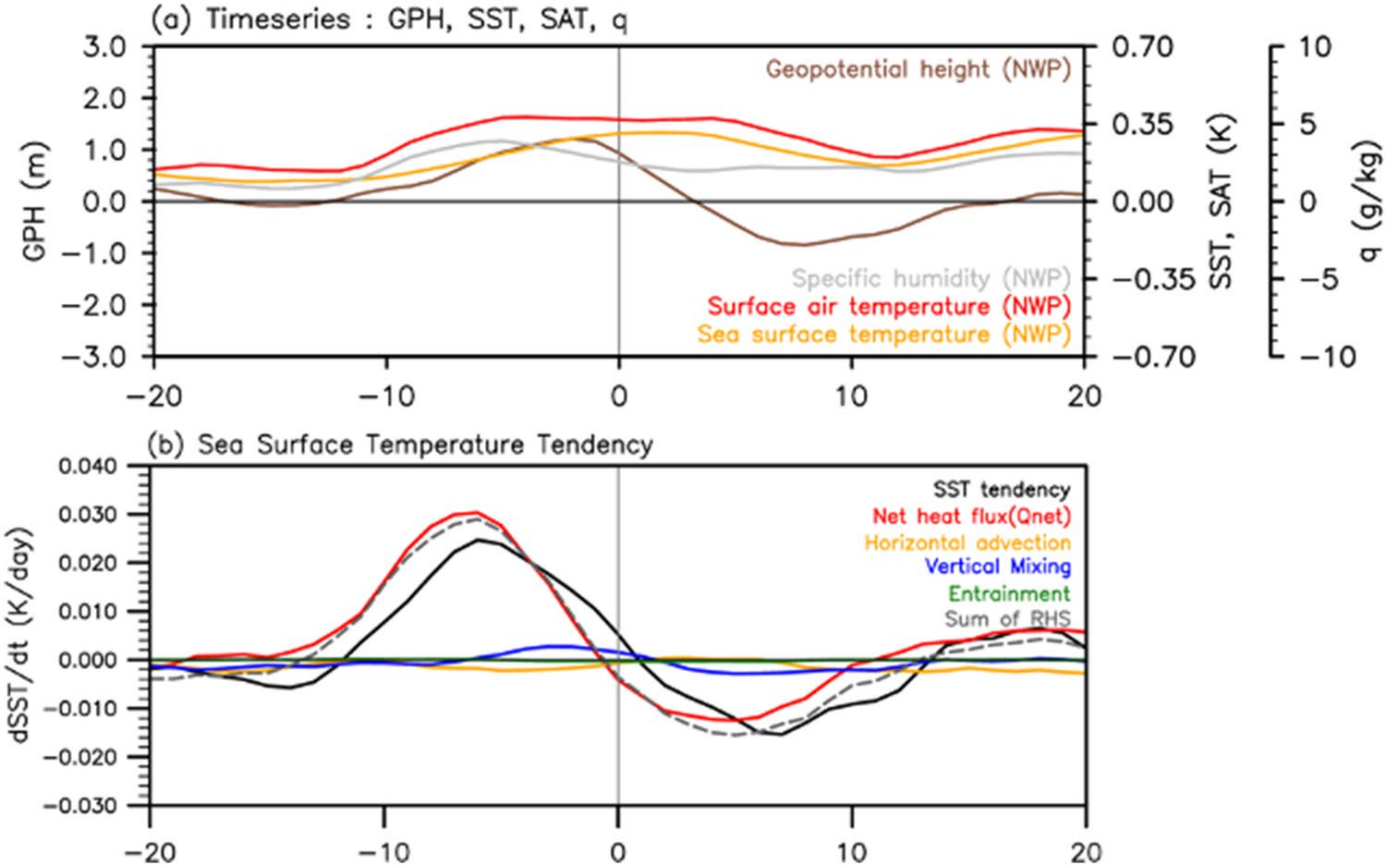
(**a**) Composite time series of 850-hPa geopotential height, surface moisture, surface air temperature, and sea surface temperature (SST) anomalies over the Northwestern Pacific (NWP, denoted in Fig. 1a) for the 39 negative Pacific–Japan patterns from day − 20 to day + 20. (**b**) Composite time series of the observed SST anomaly tendency (black) for the negative PJ patterns and forcing terms in the mixed layer heat budget equation: horizontal advection (orange), vertical mixing (blue), entrainment (green), net heat flux (red). The gray dashed curve represents the sum of the all forcing terms. 7-day running average is applied for visual clarity of the time series.

To better understand the PJ-related temporal evolution of the SST and its associated physical processes for the NWP, daily evolutions of the 850-hPa geopotential height, surface moisture, and surface air temperature anomalies were examined with the SST (Fig. 2a) The positive geopotential height anomaly over the NWP(brown curve) forms approximately 10 days before the central date of the PJ and lasts several days before it reaches a negative value. The geopotential height anomaly over the TWP shows almost the opposite behavior (not shown), indicating that the meridional dipole in Fig. 1a has coherent variability on the intraseasonal timescale. The near-surface temperature (red curve) and moisture anomalies (gray curve) over the NWP show gentle but robust increases ~ 10 days before the central date of PJ. The increasing near-surface atmospheric temperature and moisture are physically related to warm and moist advection by southeasterly flows geostrophically driven between the geopotential dipole of the PJ (Noh et al.^26^; Supplementary Fig. S1b,c). The increases in atmospheric temperature and moisture can provide favorable conditions for the SST increase by modulating the ocean–atmosphere flux exchange. The SST also showed a gradual increase during the period, with a slight delay compared with the surface air temperature. This delay suggests that atmospheric changes may serve as forcings for the subsequent SST increase, which will be further discussed using a mixed layer heat budget calculation.

The SST tendency of the East Asian coast, including the Kuroshio extension (gray box in Fig. 1b), was diagnosed using a mixed layer heat budget equation (Fig. 2b; see “Methods” for the equation). The observed SST tendency (black curve) can be resolved into the sea surface net heat flux (red curve), averaged horizontal advection (yellow curve), vertical mixing (blue curve), and entrainment at the mixed layer (green curve). The sum of the forcing terms (gray curve) agreed well with the observed tendency (black curve), indicating that the heat budget diagnosis was successfully conducted. The diagnosis reveals that the surface net heat flux (Q_net_) is the dominant factor for the SST tendency, which shows a gradual increase from ~ 10 days before the central date, with a maximum value at ~ 6 days (Fig. 2b). The tendency derived from the net heat flux presented almost the same temporal pattern as the geopotential height anomalies over the NWP. This resemblance implies that the high-pressure system associated with the negative PJ pattern provides favorable conditions for enhanced surface net heat flux and increasing SST.

**Figure 3:**
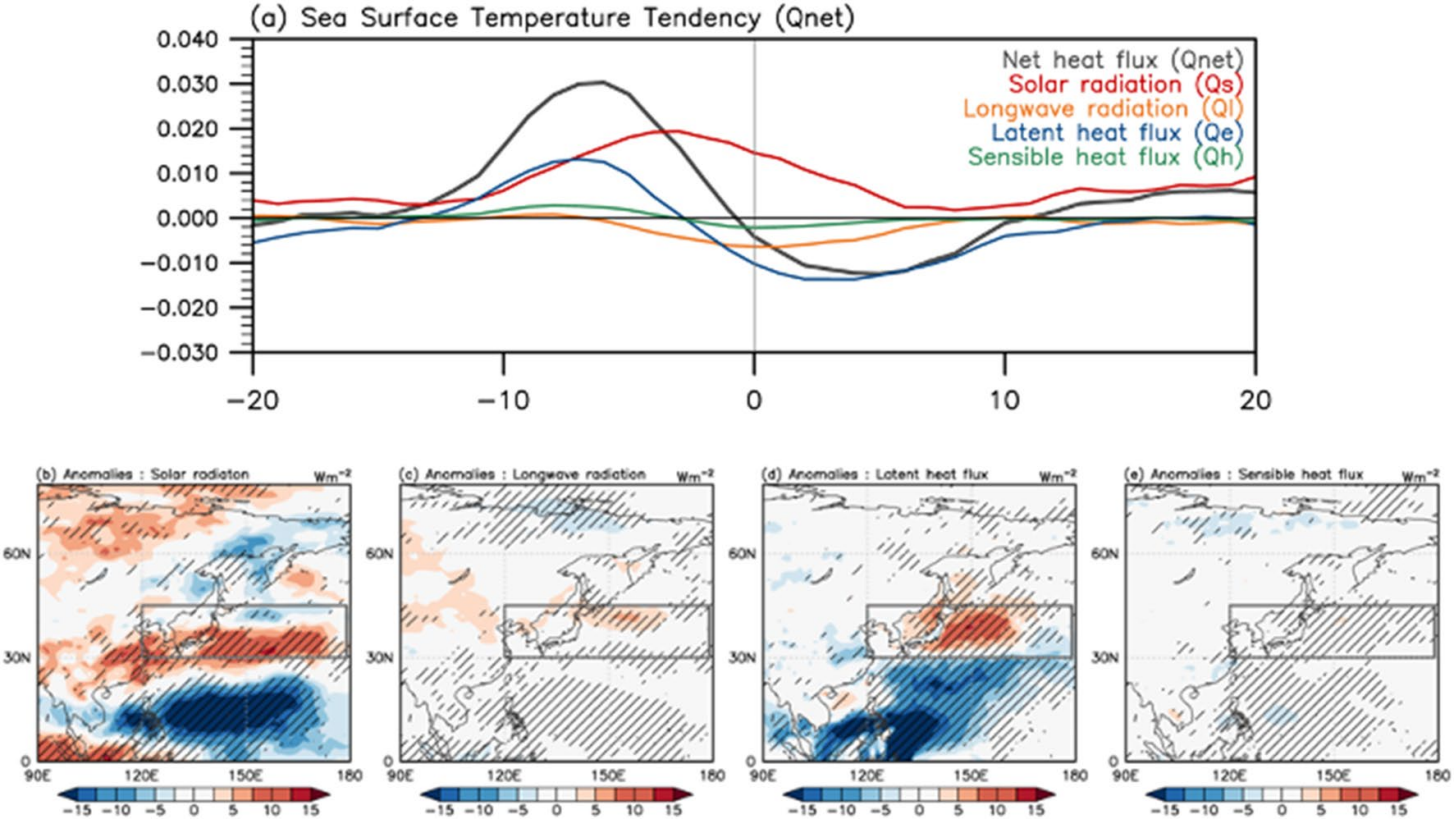
(**a**) Same as Fig. 2b, but for net heat flux and each contributing term: solar radiation (red), longwave radiation (orange), latent heat flux (blue), and sensible heat flux (green). (**b**–**e**) Horizontal distribution of each flux anomaly averaged from day − 10 to onset date (day 0). The dashed areas denote statistically significant regions at 95% confidence level based on a two-tailed Student’s *t*-test.

To examine the physical processes related to sea surface warming during a negative PJ event, we further decomposed the net surface heat flux into individual physical components. Figure 3a shows the daily time series of heat flux decomposed into solar radiation (red curve), longwave radiation (orange curve), latent heat flux (blue curve), and sensible heat flux (green curve). Figure 3b–e presents the horizontal distribution of each physical term averaged from − 10 days to the central date of the PJ. The decomposed time series revealed that solar radiation and latent heat flux were the main contributors to SST warming. Solar radiation, which plays the most dominant role in SST warming (Fig. 3a), coincides well with the high-pressure anomaly in the NWP, implying that increased insolation is related to cloud reduction in high-pressure regions. The total cloud cover showed a consistent distribution in the region (Fig. 1d). It is worth noting that solar radiation continues to heat the SST over a significantly large area, even beyond the central date (Fig. 3b), suggesting its potential role in maintaining prolonged SST warming in the mid-latitude shown in Fig. 2a.

Meanwhile, the contribution of the latent heat flux, which is also an important factor for the SST tendency, shows a slightly different behavior that anomalously heats the SST in the early phase of PJ development (− 10 to − 3 days). This is mainly explained by the reduction in heat loss by ocean surface evaporation owing to the increase in near-surface atmospheric moisture associated with the negative PJ pattern (gray curve in Fig. 2a). Despite the continued enhancement of atmospheric moisture, the latent heat effect did not last long and rapidly decreased before the central date of the PJ (Fig. 3a). This downturn is likely related to the increasing SST and the subsequent enhancement of evaporation (i.e., ocean-to-atmosphere latent heat flux). Although SST tendencies are modulated by the complex interactions of ocean–atmosphere processes, it is clear that the decrease in ocean surface evaporation in the early phase of the negative PJ event contributes to SST warming over the East Asian coast. The contributions of longwave radiation and sensible heat flux to SST tendencies were secondary with respect to PJ events.

**Figure 4:**
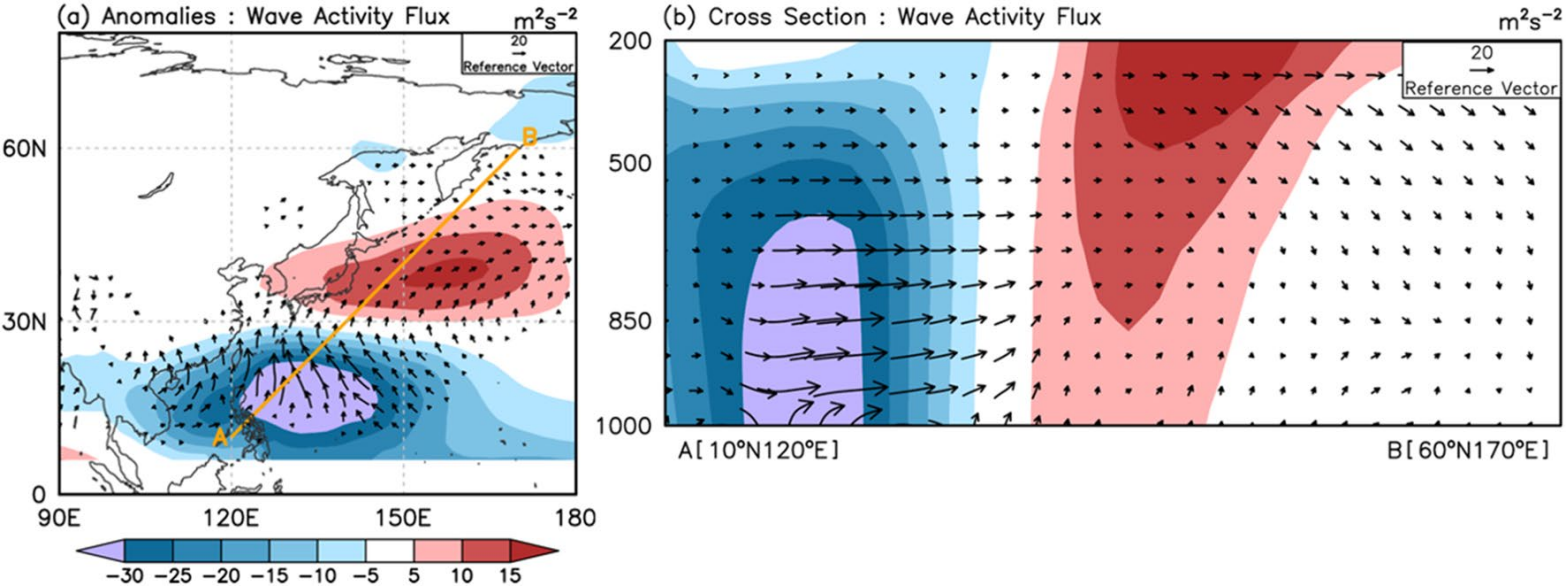
Composite map of (**a**) the 850-hPa stream function (shading; 10^6^ m^2^ s^−1^) and wave activity flux (vector; m^2^ s^−2^) anomalies for the negative Pacific–Japan (PJ) pattern from day − 10 to onset date (day 0) and (**b**) their vertical structure along the cross section A-B [from (10 N, 120 E) to (60 N,170 E)].

We examined the atmospheric PJ teleconnection pattern and found that it has suitable physical structures to facilitate SST warming over the NWP. The PJ pattern as a thermally driven Rossby wave originating from the TWP modifies mid-latitude circulation by propagating the wave poleward at the middle troposphere. The wave activity flux and stream function during the negative PJ event (Fig. 4) show the pathway of energy propagation and its impact. Owing to the mean flow structure of the NWP, the PJ pattern has a barotropic–baroclinic coupling structure (Fig. 4b) that can be maintained further through wave-mean flow interactions^32^. In addition, the deep vertical (high-pressure) structure maintained over the mid-latitude can induce stronger insolation in the upper troposphere and enhance moisture advection in the lower troposphere.

## Discussion

**Figure 5:**
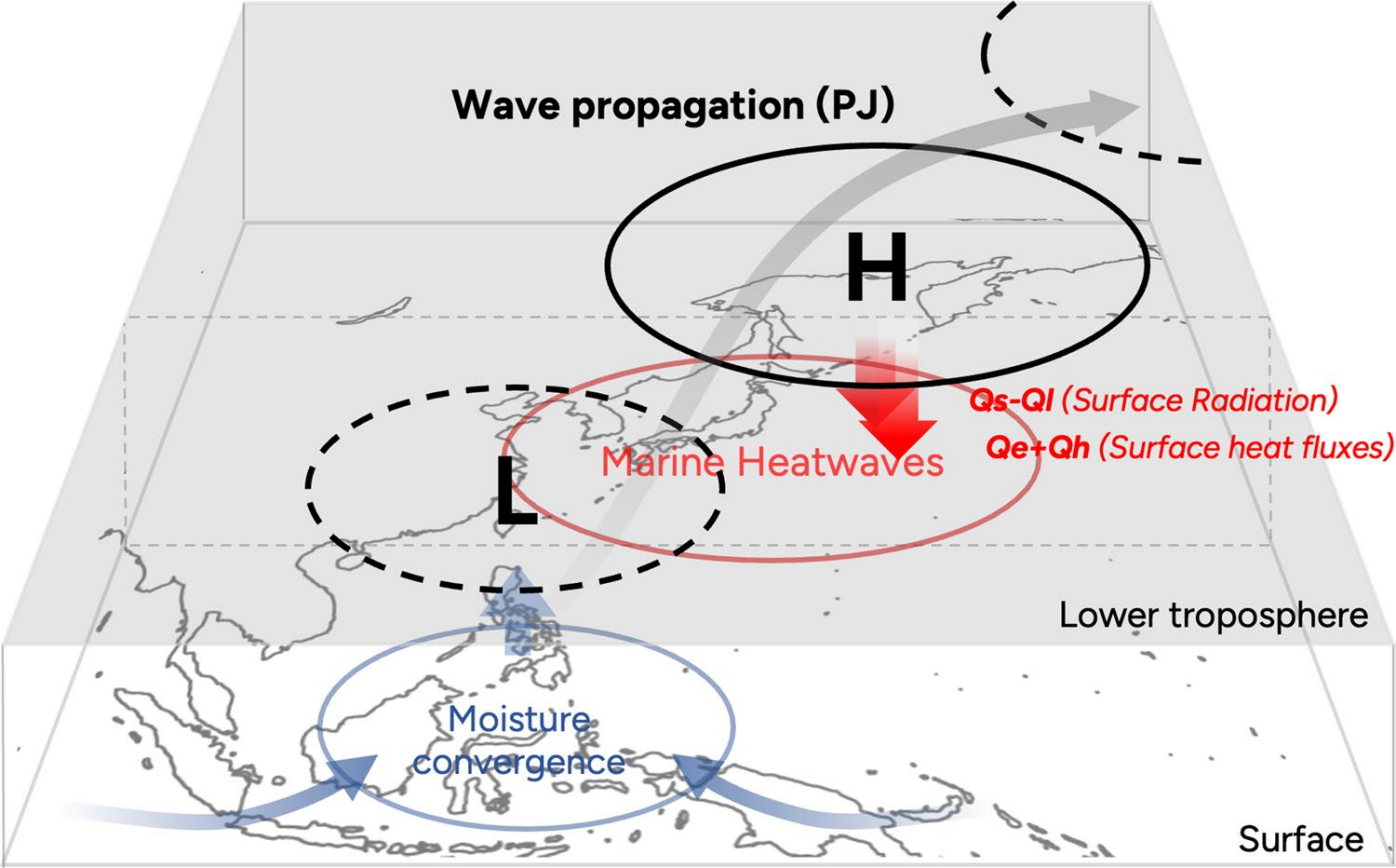
A schematic diagram of the negative Pacific–Japan (PJ) pattern and its impact on atmospheric circulation and sea surface temperature. The thick and black arrow denotes the propagation of PJ pattern (Rossby wave), and thin black contour represent related anomalous atmospheric circulation.

This study analyzed the evolution of the PJ-related sea surface warming over the NWP on an intraseasonal timescale. The key processes are enhanced solar radiation and atmospheric–ocean flux changes owing to the atmospheric PJ pattern. The PJ-related processes are summarized schematically in Fig. 5. Throughout the summer season, a substantial convergence of moisture takes place in the TWP, which is adequate to initiate deep convection (see Supplementary Fig. S4). The PJ pattern is a thermally driven Rossby wave caused by deep convection over the TWP. This Rossby wave propagates northward along southwesterly winds in the lower troposphere, forming a notable anticyclonic circulation over the Northwestern Pacific. The weather map simply appears as a northwestward extension of the NPSH, which typically leads to notable surface/sea surface warming in South Korea and southern Japan. We reported this set of processes as atmospheric pathways that trigger MHWs along the East Asian coast.

The composite anomalies based on the EOF analysis also presents the overall identical result that the negative PJ pattern is strongly correlated with sea surface warming over the NWP subregion in the vicinity of the Kuroshio current and its extension (not shown but similar to Fig. 1b). The heat budget analysis on a daily timescale revealed the surface fluxes of solar radiation and latent heat as the principal components for elevated sea surface warming. The increased solar radiation in the high-pressure region is the primary cause of the SST change, while the increasing downward latent heat flux also plays a significant role in the SST warming during the early stages of the PJ development (5–10 days before the mature stage).

MHWs are defined as extreme sea surface warming events that can be further characterized by their duration, intensity, and rate of evolution^13,22^. Recently, Hobday et al.^50^ proposed a scaling system based on the climatological mean and the 90th-percentile threshold. It ranged from moderate (Category 1) to extreme (> 4×, Category 4), with strong (2–3×, Category 2) and severe (3–4×, Category 3) classes. We aligned the averaged SST over the East Asian region (gray box in Fig. 1b) with this scale for all of the historical negative PJ cases we examined in this study (39 cases). A larger portion of the events demonstrated above-normal SST over the East Asian coast; among them, 14 events (36%) were classified as MHWs (see Supplementary Fig. S3). Thus, the negative PJ and associated circulation are related to the increase likelihood of the East Asian MHW (see Supplementary Fig. S2).

On the other hand, East Asian MHWs also have different forms apart from the PJ pattern. For example, Lee et al.^14^ identified two primary modes of East Asian MHWs through EOF analysis, accounting for 40.5% and 16.8% of the total. The first mode is primarily associated with a "persistent ocean state," which is connected to the equatorial SST changes related to El Niño-Southern Oscillation (ENSO) during early winter. The second mode is characterized by an "abrupt atmospheric forcing" tied to the dipole atmospheric circulation originating from the subtropical western Pacific, similar to the PJ pattern. These results show closely align with our result regarding the relationship between the PJ and East Asian MHWs. It should be noted that MHWs are driven by various physical factors^16^, and recent studies underlines the significance of the oceanic processes for a comprehensive understanding of the East Asian MHW^14^. Therefore, we emphasize that this study provides one specific physical mechanism for East Asian MHWs among the numerous potential physical processes.

The overall findings of this study provide meaningful insights into the development mechanism of MHW on an intraseasonal timescale, highlighting the importance of atmospheric pathways and atmospheric and ocean interactions. Atmospheric Rossby wave trains such as the PJ^45^ patterns have a significant impact on extreme heat events in their downstream regions. The equatorial SST warming, particularly over the western Pacific warm pool, can significantly modulate those patterns by altering the convective activity^46,47^. Moreover, the changes in El Nino and Southern Oscillation (ENSO) variability exert a significant influence on the near-surface circulation over the western Pacific region^38,48,49^, which provides an important background for Rossby wave propagation. The physical processes through which atmospheric teleconnection patterns influence the occurrence of MHW in the NWP require further evaluation and documentation.

The frequency and intensity of MHWs have increased with global warming^5,7,22,50^. Under warming conditions, extreme events such as atmospheric heatwaves and MHWs often occur with changes in large-scale circulation patterns^8,9^. In particular, East Asian countries have experienced a significant increase in these extreme heat events during boreal summer, and the demand for subseasonal to seasonal prediction of heat extremes is growing. Therefore, a detailed understanding of the PJ pattern and atmosphere–ocean-coupled processes related to sea surface warming would contribute to the prediction of MHW and assist proactive preparation for future MHW events.

## Methods

### Data

The primary dataset for the atmospheric analysis is the 6-h European Centre for Medium-Range Weather Forecasts (ECMWF) Re-Analysis version 5 (ERA-5^51^) regridded into a 1.5 × 1.5 resolution. Observation sea surface and surface air temperature data were sourced from the Hadley Centre Integrated Surface Database (HadISD^52^) and International Comprehensive Ocean–Atmosphere Data Set (ICOADS^53^), spanning a 43-year period from 1979 to 2021. The surface observation data employed for result verification against reanalysis data, as shown in the Supplementary figure. In addition, a high-resolution ocean reanalysis, GLORY2V4^54^ from Mercator Ocean, was utilized to compute the sea surface heat budget. To mitigate the high-frequency variability in the sea surface heat tendency, a 7-day running average was applied for the time series.

### The Pacific–Japan pattern

The PJ pattern was derived by applying empirical orthogonal function (EOF) analysis to daily 850-hPa relative vorticity field. The region used for the EOF analysis is the East Asia covering 0°–60° N and 100°–160° E, as suggested in previous studies^32,40^. In addition, the PJ index was derived from the principal component (PC) time series of the first mode obtained through the EOF analysis.

This study also examines evolution of PJ-related features through composite analyses. The PJ cases were selected based on ± 0.5 standard deviation of the PJ index, respectively, with the peak as the central date (day 0). Furthermore, we identified the cases that the index maintains same phase more than 7 days also aparting more than 7 days each other to ensure the independence of the samples. Finally, 66 positive and 39 negative cases were obtained and used for the composite analyses. Composite analyses were conducted with respect to the central date of the PJ index. Anomaly fields were generated by subtracting the daily climatology defined over the 1979–2021 period.

### Heat budget equation

Variations in the sea surface temperature are determined by the heat balance of the surface mixed layer. The ocean mixed layer thickness is defined as the depth corresponding to the density increase compared to density at 10 m depth corresponds to a temperature decrease of 0.2 °C in local surface conditions. Moreover, the surface mixed layer heat balance was affected by horizontal advection, horizontal and vertical mixing, entrainment in the mixed layer, and surface net heat fluxes. The following equations calculate all these processes^22,43,55,56^:

$$\frac{{\partial T}_{a}}{\partial t}= -\left({u}_{a}\frac{{\partial T}_{a}}{\partial x}+{v}_{a}\frac{{\partial T}_{a}}{\partial y}\right)-\frac{1}{h}{K}_{v}{\int }_{-h}^{0}\frac{{\partial }^{2}T}{{\partial z}^{2}}dz-\frac{1}{h}\left({T}_{a}-{T}_{-h}\right)\left(\frac{\partial h}{\partial t}+{u}_{-h}\frac{\partial h}{\partial x}+{v}_{-h}\frac{\partial h}{\partial y}+{w}_{-h}\right)+\frac{{Q}_{net}-{Q}_{-h}}{{\rho c}_{p}h}$$

where T, u, and v are the temperature and horizontal components of the velocity, respectively. ρ, h, c_p_, and Kv are the mean density, mixed layer depth, specific heat capacity of sea water, and vertical eddy diffusivity, respectively. The suffix a indicates a vertically averaged quantity over the mixed layer, and the suffix -h indicates the quantity at the base of the mixed layer. Here Q_net_ denotes the net surface heat flux, which is positive into the ocean, and Q_-h_ is the attenuated downward shortwave radiation at mixed layer depth.

In this study, the mean density ρ and specific heat capacity c_p_ are assumed to be a constant, 1025 kg m^−3^ and 3940 J kg^−1^ K^−1^, and vertical eddy diffusivity is 10^−4^ m^2^ s^−1^, respectively. The downward shortwave radiation at the base of the mixed layer Q_-h_ is based on an assumption of exponential decay with depth^57,58^.

$${Q}_{-h}={Q}_{0}\left[{Re}^{\left(-h/{\gamma }_{1}\right)}+\left(1-R\right){e}^{\left(-h/{\gamma }_{2}\right)}\right]$$

where Q_0_ is the downward shortwave radiation at the sea surface; and R, r_1_, and r_2_ are constants depending on water quality. In general, Jerlov water type 1 is used to calculate the attenuation quantity (Jerlov 1968). Thus, values for R (0.77), r_1_ (0.35), and r_2_ (23.0) are used.

### Supplementary Information

Supplementary Information.

## Data Availability

Copernicus Climate Change Service (C3S) (2017): ERA5: Fifth generation of ECMWF atmospheric reanalyses of the global climate. Copernicus Climate Change Service Climate Data Store (CDS), date of access. https://cds.climate.copernicus.eu/cdsapp#!/home. Met Office Hadley Centre; National Centers for Environmental Information—NOAA (2020): HadISD: Global sub-daily, surface meteorological station data, 1931–2019, v3.1.0.2019f. Centre for Environmental Data Analysis, date of citation. https://catalogue.ceda.ac.uk/uuid/e488dccd09e1446d90978b75036475e2. This study has been conducted using E.U. Copernicus Marine Service Information; https://doi.org/10.48670/moi-00024.
