# Supplementary material for: Atmospheric pathway of marine heatwaves over the Northwestern Pacific
Source: Sci Rep. 2023 Dec 20;13:22821. doi: 10.1038/s41598-023-49833-4 (PMC10739699; doi:10.1038/s41598-023-49833-4)
Supplement: Supplementary file 1 — Supplementary Information. [file 41598_2023_49833_MOESM1_ESM.pdf]

Supplementary Figure

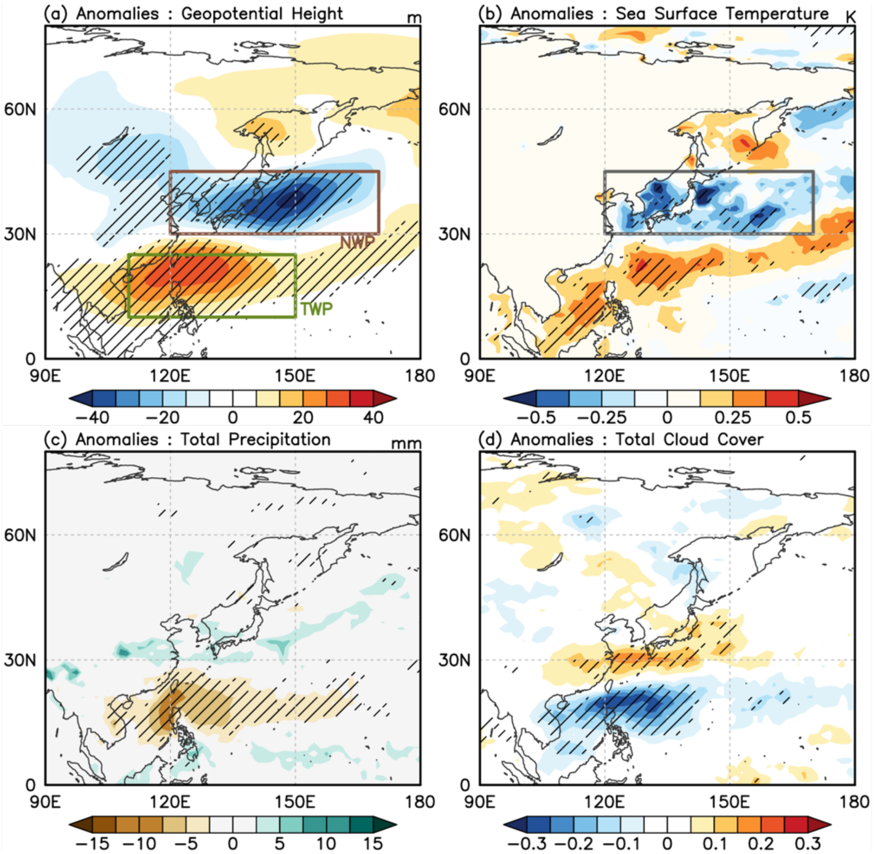

Supplementary Figure 1. Same as in Fig. 1, but for positive phase of the PJ patterns.

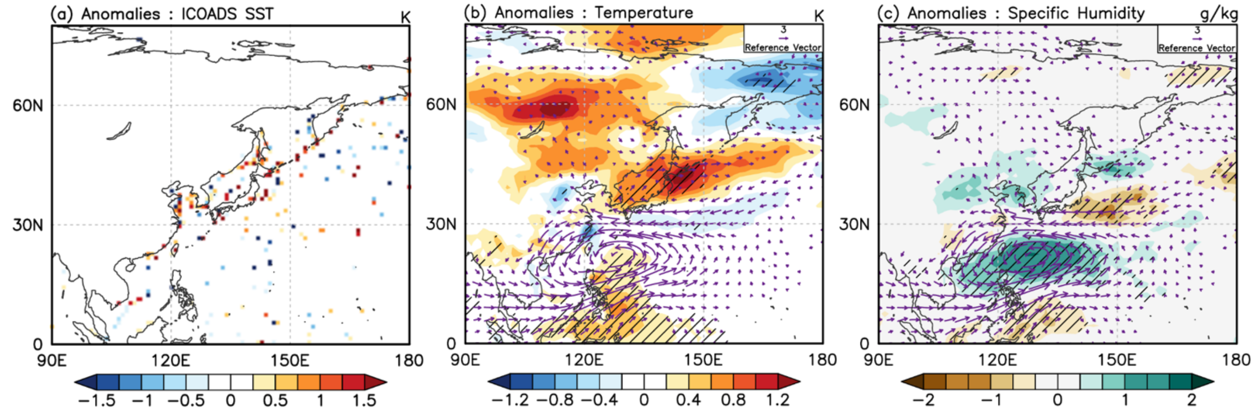

Supplementary Figure 2. Composite distribution of (a) in-situ observations (ICOADS) of sea surface temperature (K), and ERA5 (b) 850-hPa temperature (K) and (c) specific humidity (g/kg) for the negative PJ patterns. The vectors in (b, c) indicates horizontal wind anomalies during the negative PJs. The dashed areas denote statistically significant area at 95% confidence level based on a two-tailed Student's *t*-test.

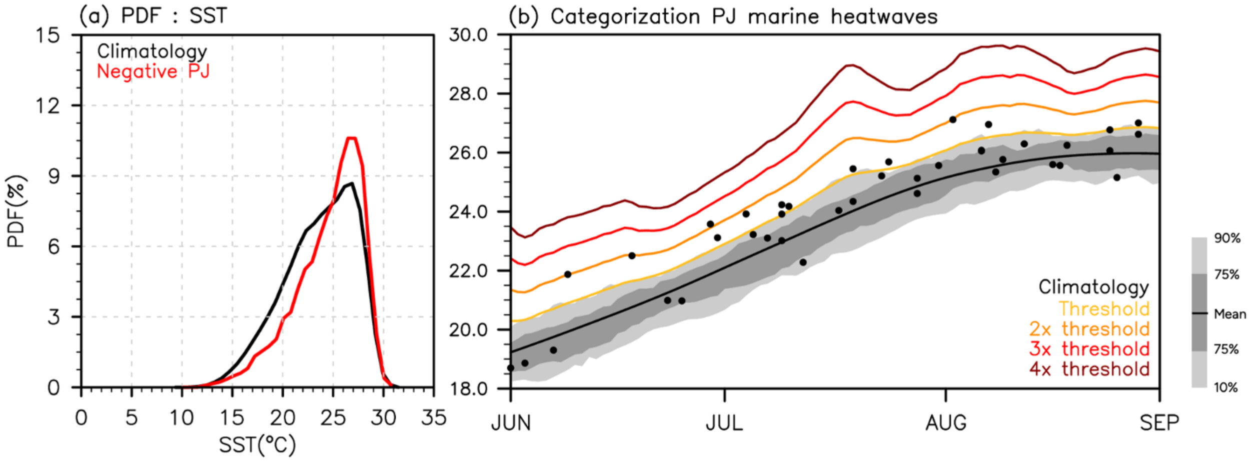

Supplementary Figure 3. (a) Probability density function of SST averaged over the East Asian domain (Grey box in Fig. 1b) for the negative PJs and climatology. (b) Categorization of PJ-related MHWs along with the long-term daily climatology (black line) and the 90th percentile climatology (yellow line; used as MHW threshold) over East Asia (Grey box in Fig. 1b). Multiples of the 90th percentile difference from the climatology value are also shown to visualize the categories defined in Hobday et al. (2018). The shading represents the 75th and 90th percentiles for the East Asian SST, respectively.

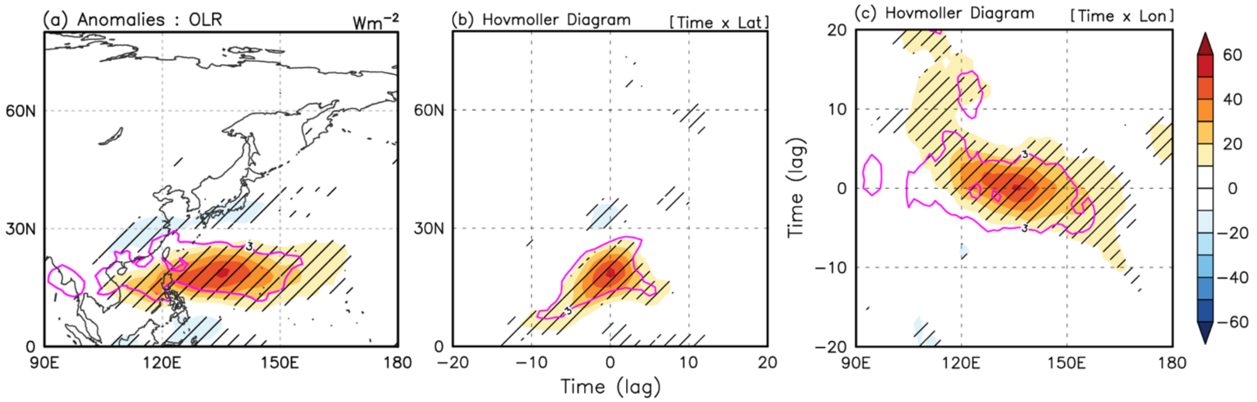

Supplementary Figure 4. (a) Composite distribution of outgoing longwave radiation (shading; W m^{-2}) and vertically integrated moisture convergence (contour; 10^6 s^{-1}) anomalies for the negative PJ. (b, c) Temporal evolution of the anomalies in time-latitude (time-longitude) diagrams (the reference latitude and longitude are 20°N, 135°E, respectively). The time axis represents days with respect to the day of the PJ event (lag 0). The dashed areas denote statistically significant anomalies at 95% confidence level based on a two-tailed Student's *t*-test.
